# Chronic Effects of a *Salmonella* Type III Secretion Effector Protein AvrA *In Vivo*

**DOI:** 10.1371/journal.pone.0010505

**Published:** 2010-05-05

**Authors:** Rong Lu, Shaoping Wu, Xingyin Liu, Yinglin Xia, Yong-guo Zhang, Jun Sun

**Affiliations:** 1 Department of Medicine, University of Rochester, Rochester, New York, United States of America; 2 Department of Biostatistics and Computational Biology, University of Rochester, Rochester, New York, United States of America; 3 Department of Microbiology and Immunology, University of Rochester, Rochester, New York, United States of America; 4 Wilmot Cancer Center, University of Rochester, Rochester, New York, United States of America; Charité-Universitätsmedizin Berlin, Germany

## Abstract

**Background:**

*Salmonella* infection is a common public health problem that can become chronic and increase the risk of inflammatory bowel diseases and cancer. AvrA is a *Salmonella* bacterial type III secretion effector protein. Increasing evidence demonstrates that AvrA is a multi-functional enzyme with critical roles in inhibiting inflammation, regulating apoptosis, and enhancing proliferation. However, the chronic effects of *Salmonella* and effector AvrA *in vivo* are still unknown. Moreover, alive, mutated, non-invasive *Salmonella* is used as a vector to specifically target cancer cells. However, studies are lacking on chronic infection with non-pathogenic or mutated *Salmonella* in the host.

**Methods/Principal Findings:**

We infected mice with *Salmonella* Typhimurium for 27 weeks and investigated the physiological effects as well as the role of AvrA in intestinal inflammation. We found altered body weight, intestinal pathology, and bacterial translocation in spleen, liver, and gallbladder in chronically *Salmonella*-infected mice. Moreover, AvrA suppressed intestinal inflammation and inhibited the secretion of cytokines IL-12, IFN-γ, and TNF-α. AvrA expression in *Salmonella* enhanced its invasion ability. Liver abscess and *Salmonella* translocation in the gallbladder were observed and may be associated with AvrA expression in *Salmonella*.

**Conclusion/Significance:**

We created a mouse model with persistent *Salmonella* infection *in vivo*. Our study further emphasizes the importance of the *Salmonella* effector protein AvrA in intestinal inflammation, bacterial translocation, and chronic infection *in vivo*.

## Introduction

Salmonellosis is one of the most common enteric infections in the United States. In addition to the public health concern of *Salmonella* outbreaks, *Salmonella* colonization and infection can be chronic [1] and increase the risk of other gastrointestinal (GI) diseases, including chronic inflammation and gallbladder cancer [2], [3]. A recent population-based cohort study demonstrates an increased risk of inflammatory bowel diseases in individuals with *Salmonella* infection [2]. Chronic *Salmonella* colonization in intestine increases the risk of the intestinal fibrosis in mice [1]. Although the pathogenesis of *Salmonella*-induced diarrhea and inflammation has been extensively studied [4], how *Salmonella* regulates the host responses in the long term remains unknown.

Increasing evidence links some *Salmonella* species to carcinogenesis, whereas others appear promising in the diagnosis, prevention, or treatment of cancers [5]. *Salmonella* and its derivatives prefer solid tumors over normal tissue in animal models [6], [7]. Alive, mutated, non-invasive *Salmonella* has been used as a vector to specifically target cancer cells [8]. However, there have been no studies on chronic infection with non-pathogenic or mutated *Salmonella* in the host.

Bacteria can modulate host physiology by secreting bacterial proteins, called effectors, through a type III secretion system [9], [10], [11], [12]. These 'molecular syringes' inject effectors directly into the host cell, whereupon they manipulate host cell functions [13], [14], [15], [16]. AvrA is a bacterial effector protein existing in both *Salmonella* and *E. coli* strains [17]. Increasing evidence demonstrates that AvrA is a multi-functional protein with critical roles in inhibiting inflammation, regulating epithelial apoptosis, and enhancing proliferation [17], [18], [19], [20], [21], [22], [23]. However, the chronic effects of *Salmonella* AvrA *in vivo* are unexplored.

We colonized the mouse intestine with *Salmonella* Typhimurium for 27 weeks. To determine whether AvrA is responsible for the chronic intestinal inflammation, we focused on bacterial strains with parental PhoP^c^, PhoP^c^ AvrA mutant (AvrA^−^), and PhoP^c^ AvrA^−^ with a complemented plasmid encoding AvrA (PhoP^c^ AvrA^−^/AvrA^+^) *in vivo*, as we used in previous studies [20], [21], [22], [24], [25]. We observed the bacteria in intestine, the translocation of *Salmonella* in liver, gallbladder, and spleen, and inflammatory responses of intestinal mucosa over 27 weeks. We observed that AvrA persistently modulates intestinal inflammation *in vivo*. AvrA expression in *Salmonella* enhanced its invasion ability. Liver abscess and *Salmonella* translocation in the gallbladder were observed and may be associated with AvrA expression in *Salmonella*. Overall, our study emphasizes the importance of AvrA in *Salmonella*-induced intestinal inflammation and chronic infection *in vivo*.

## Results

### Persistence of *Salmonella* infection in the streptomycin-pretreated mouse model

**Figure 1:**
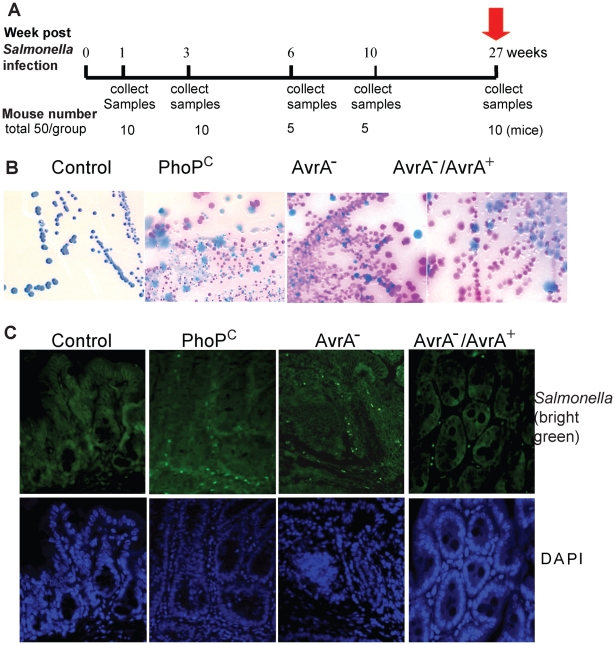
Persistent *Salmonella* infection in mice. (A) Flow chart of the experimental design. (B) Chronic *Salmonella* infection was measured by fecal matter analysis. In a BBL CHROMagar plate, intestinal *Salmonella* species appear mauve (rose to purple) in color, due to metabolic differences in the presence of selected chromogens. Other bacteria are either inhibited or produce blue-green or colorless colonies. Experimental groups: Control, normal wild-type mice; PhoP^c^, mice infected with *Salmonella* with normal AvrA expression; PhoP^c^AvrA^−^, mice infected with an AvrA^−^ mutant derived from PhoP^c^; PhoP^c^AvrA^−^/AvrA^+^, mice infected with PhoP^c^AvrA^−^ containing a complementary plasmid encoding AvrA. (C) Location of *Salmonella* in mouse intestine using immunofluorescence staining. Green staining in the mouse intestine shows the invasion of *Salmonella* in intestinal mucosa at 27 weeks postinfection.

*S.* Typhimurium infection can last over several months *in vivo* [1]. However, it is uncertain whether *Salmonella* PhoP^c^ and its derived mutations are able to colonize the intestine *in vivo*. We tested AvrA^+^ and AvrA^−^ bacterial colonization in mice post infection for 27 weeks. We collected tissue samples at 1, 3, 6, 10, and 27 weeks postinfection (Fig. 1A). First, we confirmed the colonization of *Salmonella* in the intestine by fecal bacterial culture. *Salmonella* existence and AvrA expression in the gut flora were also confirmed by *Salmonella* culture (Fig. 1B). Immunofluorescence analysis of intestinal tissue samples showed the invasion of *Salmonella* in intestinal mucosa at 27 weeks postinfection (Fig. 1C). PCR of AvrA further confirmed that AvrA was expressed in the mouse intestine and feces (data not shown). These data demonstrate that one gavage of *Salmonella* was sufficient to induce persistent *Salmonella* colonization in the intestine for 27 weeks in the streptomycin-pretreated mouse model.

### Chronic *Salmonella* infection retards body weight gain in the streptomycin-pretreated mouse model

**Figure 2:**
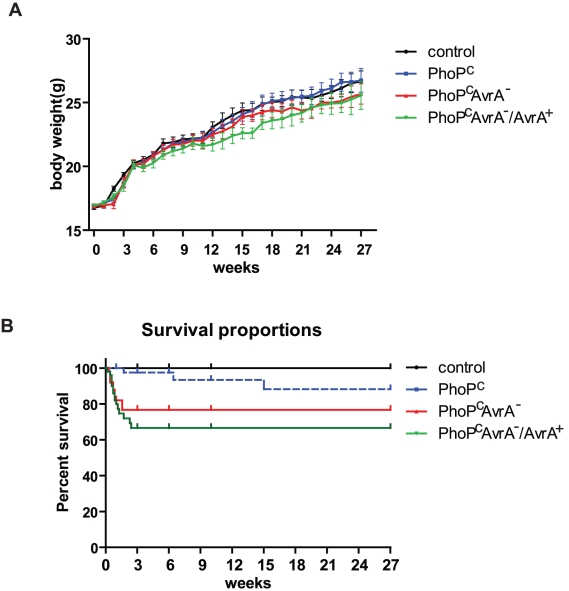
Physiologic changes after *Salmonella* infection. (A) Changes in body weight after *Salmonella* infection. (B) The survival rate of mice after Salmonella Typhimurium infection for 27 weeks. N = 50 mice in each group. Mice were infected with indicated Salmonella Typhimurium strains for 27 weeks. Mice were weighted every week. If a mouse showed indications of aspirated fluid or significant body weight loss (10% or more), and did not die immediately, the mouse was humanely euthanized. The protocol was approved by the University of Rochester University Committee on Animal Resources (UCAR).

Bacterial infection may induce weight loss in mice. We monitored changes in body weight in the control and *Salmonella*-infected mice for 27 weeks (Fig. 2A and Table 1). From week 0 to week 27, overall weight increased for all these four groups, that is, there are time effects among these four groups. The increasing rates were significantly different between control and *Salmonella*–infected group, PhoP^c^, Phop^c^AvrA^−^, or Phop^c^AvrA^−^/Avra^+^, although there were no significant differences among all these three *Salmonella*–infected groups. During the beginning 2 weeks after *Salmonella* infection, the mice body weights increasing rates from the three *Salmonella*–infected groups all slowed down, until week 2 or 3, the average weight of all the three *Salmonella*–treated groups were significantly lower that the control group at the .05 significance level. For PhoP^c^ vs. control, the difference started at week 2, continued until week 9; for Phop^c^AvrA^−^, vs. control, the difference started at week 2, lasted to week 6; as for Phop^c^AvrA/Avra^+^ vs. Control, the difference started at week 3, but over the whole treatment period until week 27. In other words, the body weight loss in the three *Salmonella*–infected mice need 4 to 7 weeks, even 24 weeks to catch up.

**Table 1:** Body weight of mice infected with *Salmonella* for 27 weeks.

Groups	0 week	1 week	2 weeks	3 weeks	4 weeks	5 weeks	6 weeks
Control	16.78±1.05	16.91±1.28	18.26±1.23	19.31±1.26	20.26±1.15	20.52±1.15	20.92±1.13
PhoP^c^	16.89±1.03	17.14±1.19	17.43±1.63	18.68±1.78	20.05±1.11	20.33±1.22	20.84±1.02
PhoP^c^AvrA^−^	16.91±1.12	16.96±1.39	17.06±2.14	18.71±2.18	20.15±1.56	20.21±1.57	20.83±1.39
PhoP^c^AvrA^−^/AvrA^+^	16.94±0.96	17.17±1.02	17.63±1.42	18.44±2.03	20.09±1.17	19.90±1.18	20.27±1.49

	7 weeks	8 weeks	9 weeks	10 weeks	11 weeks	12 weeks	13 weeks
Control	21.80±1.46	21.89±1.70	22.12±1.40	22.18±1.55	22.24±1.55	23.08±1.43	23.58±2.11
PhoP^c^	21.30±1.62	21.79±1.54	21.91±1.53	22.12±1.51	22.21±2.04	22.68±1.66	23.17±2.26
PhoP^c^AvrA^−^	21.27±1.61	21.71±1.49	21.74±1.15	22.03±1.59	22.00±1.60	22.50±1.31	22.75±1.60
PhoP^c^AvrA^–^/AvrA^+^	20.90±1.04	21.20±1.15	21.40±1.07	21.80±1.48	21.60±0.55	21.70±1.10	22.00±1.41

	14 weeks	15 weeks	16 weeks	17 weeks	18 weeks	19 weeks	20 weeks
Control	24.00±2.00	24.38±2.01	24.41±1.88	24.91±1.85	25.04±1.36	25.11±1.49	25.44±1.92
PhoP^c^	23.56±2.12	24.08±2.25	24.38±2.85	24.81±3.10	25.13±2.39	25.22±2.52	25.36±2.36
PhoP^c^AvrA^−^	23.13±1.72	23.88±1.64	24.00±1.31	24.25±1.67	24.41±1.67	24.29±1.74	24.63±1.60
PhoP^c^AvrA^−^/AvrA^+^	22.40±1.34	22.60±0.97	22.60±0.89	23.40±1.14	23.60±1.47	23.72±1.58	24.00±1.87

	21 weeks	22 weeks	23 weeks	24 weeks	25 weeks	26 weeks	27 weeks
Control	25.41±1.90	25.34±2.37	25.58±1.58	25.83±1.73	26.13±2.35	26.52±2.57	26.65±2.87
PhoP^c^	25.47±2.03	25.53±2.64	25.93±2.43	26.13±2.75	26.60±2.75	26.62±2.96	26.75±3.58
PhoP^c^AvrA^−^	24.38±1.85	24.55±2.21	25.00±1.41	25.00±1.20	25.13±1.46	25.46±2.37	25.68±2.26
PhoP^c^AvrA^−^/AvrA^+^	24.18±1.55	24.62±1.65	24.80±1.48	24.92±1.89	24.96±1.66	25.24±2.64	25.56±2.43

### Various survival rates in mice infected with different *Salmonella* strains

The PhoP^c^ strain was derived from pathogenic *Salmonella* SL14028 and was used as a non-pathogenic *Salmonella* strain in previous short-term intestinal inflammation studies [26] [21], [24], [25] . However, we found that PhoP^c^ *Salmonella* infection could be lethal in mice (Fig. 2B). Survival analysis showed that most deaths occurred at 3 weeks postinfection. After the acute infection stage, mice survived with persistent *Salmonella* detected in intestine for 27 weeks. The PhoP^c^ *Salmonella* infection group had the highest survival rate, 90%, whereas AvrA^−^ had a survival rate of 77%, and that of PhoP^c^ AvrA^−^/AvrA^+^ was 68%. These data indicate that complementary plasmid-expressed AvrA in the AvrA^−^ strain was not able to increase the survival rate during long-term infection.

### Intestinal shortening induced by *Salmonella in vivo*

**Figure 3:**
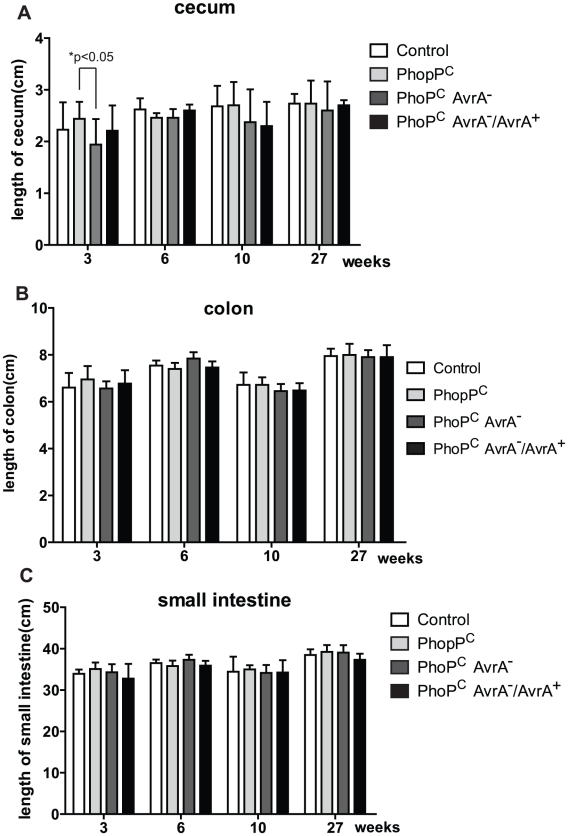
Intestinal shortening induced by *Salmonella in vivo*. (A) Length of ceca. (B) (C) Lengths of small intestine and colon. N = 50 mice in each group and 5–10 mice at each time point.

Cecal shortening and inflammation are the key features in the *Salmonella*-colitis mouse model [27]. We found that the lengths of ceca in groups infected with *Salmonella* were decreased compared to the mice without *Salmonella* infection. At 3 weeks postinfection, significant shortening of ceca was found in the AvrA^−^ infection group compared to the PhoP^c^ group (Fig. 3A). Inflammation and gross pathology are focused on the cecum, with some inflammation in the contiguous colon in the streptomycin-pretreated *Salmonella* colitis model [28]. We also measured the lengths of small intestine and colon but did not find significant differences (Fig. 3B and C).

### Pathologic changes caused by chronic *Salmonella* infection in streptomycin-pretreated mice

**Figure 4:**
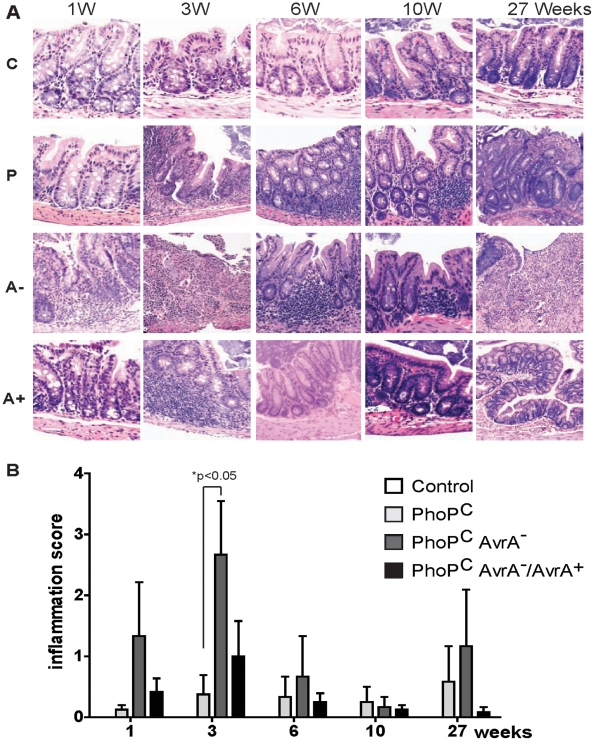
Pathologic changes caused by the chronic *Salmonella* infection in streptomycin-pretreated mice. (A) H & E staining of cecum in mice infected with *S*. Typhimurium at 1, 3, 6, 10 and 27 weeks postinfection compared to the wild-type mice (Control). C: Control; P: PhoP^c^; A-: PhoP^c^AvrA^−^; and A+: PhoP^c^AvrA^−^/AvrA^+^. (B) Histological score of cecal inflammation. Data are from three mice in each group at each time point. *, p<0.05.

We investigated the pathologic changes caused by chronic *Salmonella* infection in streptomycin-pretreated mice. H&E staining showed severe intestinal inflammation in mice infected with *Salmonella* Typhimurium AvrA^−^ at 1, 3, 6, 10, and 27 weeks postinfection compared to the wild-type mice (Fig. 4A). Next, the level of cecal inflammation was quantified histologically. After 3 week post treatment, the mean cecum histologic score for PhoP^c^AvrA^−^ group was significantly higher than that of control group; PhoP^c^AvrA^−^ group was significantly higher than that of PhoP^c^ group at .05 significance level. The mean cecum score of PhoP^c^AvrA^−^/AvrA^+^ group is lower than that of PhoP^c^AvrA^−^ group with trend evidence (p-value = .06). Overall, inflammation score in the AvrA^−^-infected mice was higher than the PhoP^c^- or PhoP^c^ AvrA^−^/AvrA^+^–infected mice at 3 weeks postinfection (Fig. 4B). These data indicate that AvrA expression consistently suppressed intestinal inflammation.

### Changes in the weights of spleen and liver post–*Salmonella* infection

**Figure 5:**
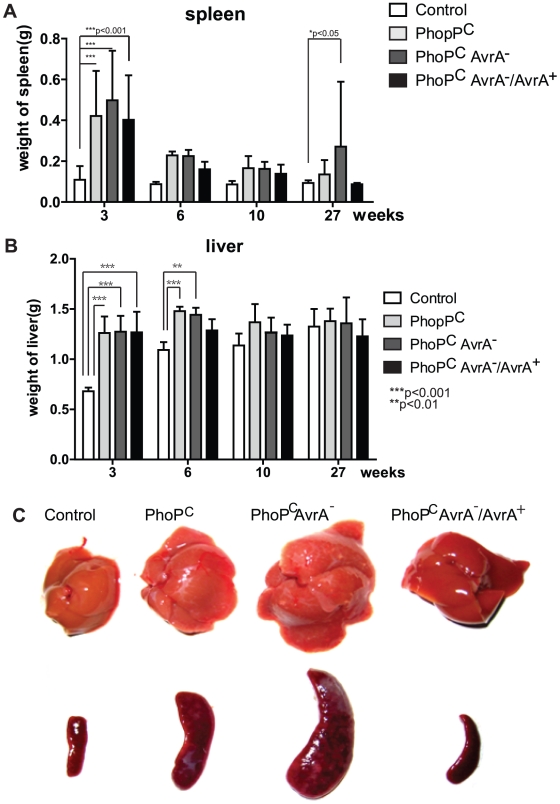
Liver and spleen affected by *Salmonella* infection *in vivo*. (A) Weights of spleens in mice after *Salmonella* infection for 27 weeks. (B) Weights of livers in mice after *Salmonella* infection for 27 weeks. Data are from 5–10 mice in each group at each time point. **, p<0.01. ***, p<0.001. (C) Enlarged livers and spleens after *Salmonella* infection for 27 weeks.

Both liver and spleen are involved in *Salmonella* infection. We measured the weights of spleens and livers after various lengths of infection. At week 3, significant spleen weight increase was found in all the *Salmonella* infection groups. At week 27, the AvrA^−^ infection group had significant heavier spleens than the control group without *Salmonella* (Fig. 5A). Similarly, the infection groups had significantly heavier livers than those in the untreated control group at week 3. At week 6, both PhoP^c^ and AvrA^−^ infection significantly increased liver weight compared to the control (Fig. 5B). Enlarged livers and spleens were found in the *Salmonella*-infected groups. Fig. 5C shows the liver and spleens from mice at 27 weeks postinfection.

### *Salmonella* translocation in spleen, liver, and gallbladder *in vivo*

**Figure 6:**
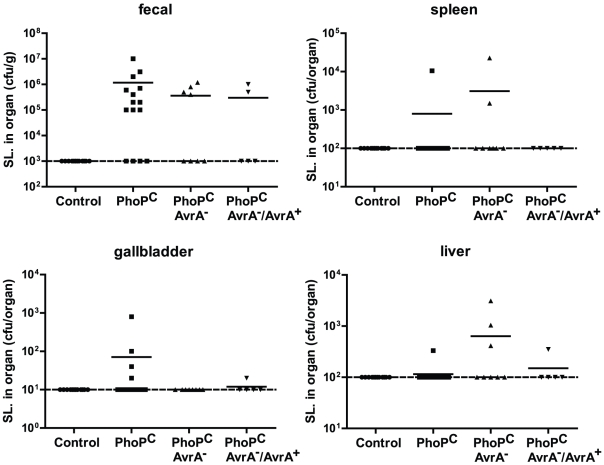
Numbers of *Salmonella* in different organs at 27 weeks postinfection *in vivo*. (A) Feces. (B) Spleen. (C) Gallbladder. (D) Liver. N = 5 mice in each group.

*Salmonella* translocation is commonly reported in *Salmonella*-infected patients and animals [29] [30], [31]. Using our chronic *Salmonella*-infected mouse model, we further observed the *Salmonella* burden in various organs over 27 weeks. The numbers of CFU were determined by plating on MacConkey agar plates for *Salmonella* colonies. Fecal cultures showed the persistence *Salmonella* in the intestine for 27 weeks. Our data also showed *Salmonella* translocation in spleen, liver, and gallbladder (Fig. 6). The PhoP^c^ infection group had the highest bacterial number in the gallbladder. The overall physical features of PhoP^c^-infected mice were better than AvrA^−^, even though extensive *Salmonella* invasion was identified in the intestine and other organs.

### Distribution of *Salmonella* in gallbladder

**Figure 7:**
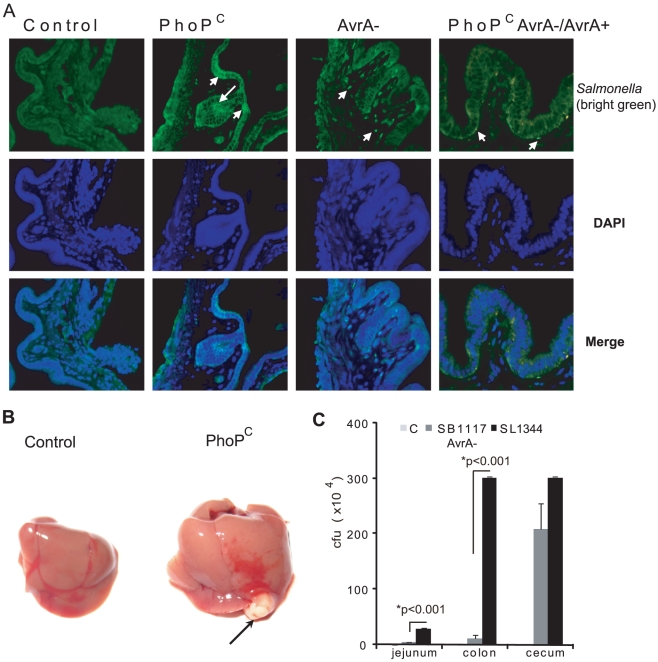
*Salmonella* translocation and infection in gallbladder and liver. (A) *Salmonella* distribution in the gallbladder at 27 weeks after *Salmonella* infection. *Salmonella*: green staining (indicated with white arrows); nucleus: blue. (B) Liver abscess found in the PhoP^c^-infected mouse (indicated with an arrow). (C) Numbers of *Salmonella* in intestine at 18 hours post-infection.

The gallbladder is a permissive site for *Salmonella*. *Salmonella* infects the single epithelial cell layer of the gallbladder but rarely translocates to the underlying lamina propria [32]. Therefore, we investigated the distribution of *Salmonella* in the gallbladder using immunostaining. Our data indicate the presence of *Salmonella* in the gallbladder at 27 weeks postinfection (Fig 7A). *Salmonella* localized to the epithelial cells, which is consistent with a recent paper on *Salmonella* invasion [32]. Moreover, PhoP^c^-infected mice had more abundant *Salmonella* in the gallbladder than the other groups.

### Liver abscess induced by chronic *Salmonella* infection

Liver abscess was found in mice with chronic *Salmonella* infection (Fig. 7B). Interestingly, more liver abscesses were found in the PhoP^c^-infected group (80% of mice) than in the control mice (0%) at 6 weeks postinfection (Table 2). In contrast, no liver abscess was found in the PhoP^c^AvrA^−^-infected mice. At 10 weeks postinfection, 40% of liver abscesses was found in the PhoP^c^ and PhoP^c^AvrA^−^/AvrA^+^ groups, whereas only 20% of mice had a liver abscess in the AvrA^−^ group. These data raise the possibility that AvrA expression increases *Salmonella* infection in the liver, possibly by enhancing the translocation ability of the *Salmonella* strains.

**Table 2:** Percentage of mice with liver abscess.

	6 weeks (n = 5 mice)	10 weeks (n = 5 mice)
Control	0	0
PhoP^c^	80%	40%
PhoP^c^AvrA^−^	0	20%
PhoP^c^AvrA^−^/AvrA^+^	0	40%

### AvrA expression in *Salmonella* enhances *Salmonella* invasion

The role of AvrA in bacterial invasion is unknown. We further compared the invasion ability of *Salmonella* strains with or without AvrA expression using pathogenic *Salmonella* 1344 with AvrA and its derived mutant strain SB1117 AvrA^−^. We found that *Salmonella* 1344 with AvrA had better invasion capability than SB1117 AvrA^−^ in jejunum and colon 18 hours postinfection. The *Salmonella* number in the intestine was significantly higher in mice infected with strain SB1117 AvrA^−^ than with strain 1344 (Fig. 7C). These data indicate AvrA promotes intestinal *Salmonella* invasion *in vivo*.

### Profile of inflammatory cytokines in *Salmonella*-infected mice

**Figure 8:**
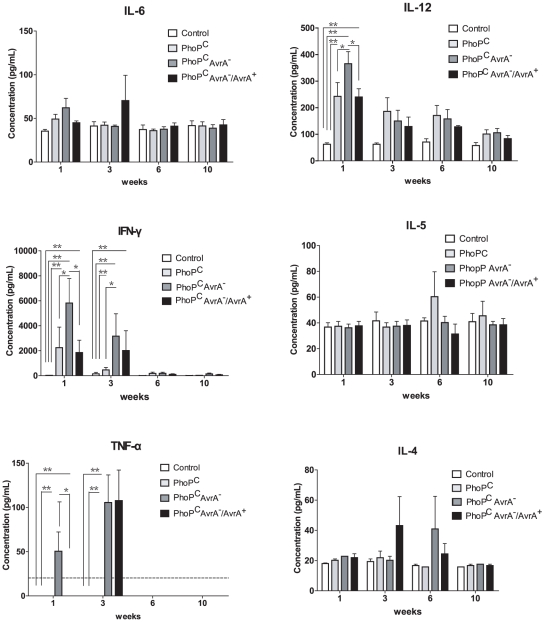
Inflammatory cytokines in chronically *Salmonella*-infected mice. The cytokines included CSF, IL-1β, IL-2, IL-4, IL-5, IL-6, IL-10, IL-12 (p40/p70), IFN-γ, and TNF-α. They were tested in mouse serum using a mouse Cytokine 10-Plex Panel. * p<0.01. ** p<0.0005. Each single experiment was assayed in triplicate. Data are means ± SD of n = 4 mice in each group.

Inflammatory cytokines play a complex role in intestinal inflammation. We then investigated the profile of cytokines in mouse serum. The cytokines we tested included Colony-stimulating factor (CSF), interleukin (IL)-1β, IL-2, IL-4, IL-5, IL-6, IL-10, IL-12 (p40/p70), interferon (IFN)- γ, and tumor necrosis factor (TNF)- α. At 1 week postinfection, significant differences in IL-12 secretion were found among PhoP^c^, AvrA^−^, and AvrA^−^/AvrA^+^. For IFN-γ, significant differences were also found at 1 and 3 weeks postinfection. TNF-α was significantly increased in the AvrA^−^ group at 1 week postinfection. At 3 weeks, both AvrA^−^ and AvrA^−^/AvrA^+^ had significantly higher TNF-α in serum, whereas there was no detectable TNF-α in the PhoP^c^-infected mice. We found no differences in IL1β or IL-4. There was no detectable IL-10 or CSF in any experimental group (Fig. 8). Overall, AvrA expression in *Salmonella* suppressed the secretion of cytokines, namely IL-12, IFN-γ, and TNF-α.

## Discussion

In the current study, we have established a chronic bacterial infected mouse model with *Salmonella* Typhimurium colonization in the mouse intestine for 27 weeks. Many researchers have taken advantage of the colitis induced by *Salmonella* Typhimurium to study the early phase of inflammation and infection. However, only a few reports have been on chronic infection *in vivo* [1]. Our model with the persistent presence of *Salmonella* AvrA^+^ and AvrA^−^ in the GI tract allows us to explore the long-term host–bacterial interaction, infection, inflammation, immunology, signal transduction, and tumorigenesis.

Moreover, we identified the long-term effects of a *Salmonella* effector protein AvrA *in vivo*. We found that AvrA consistently inhibits intestinal inflammation *in vivo*. This study provides fundamental knowledge and insight into the mechanism of how enteric bacterial protein AvrA regulates intestinal inflammation and infection over the long term. Furthermore, our data demonstrate that AvrA expression in *Salmonella* increases bacterial invasion and translocation. Bacterial infection and inflammation are known to be associated with tumorigenesis. Future studies will use colon cancer models to investigate the effects of AvrA in tumorigenesis.

Liver abscess was observed in mice with chronic *Salmonella* infection without intestinal symptoms. These data are consistent with clinical case reports on the *Salmonella* infection in human [33]. Peritoneum adhesion to the liver (peritonitis), infection in the uterine horn, otisis, and eye infection have also been found in some mice, induced by bacterial translocation. These observations have also been reported in studies of *Salmonella*-infected humans and chickens [30], [31] [34].

Liver abscess and *Salmonella* translocation in the gallbladder are associated with a mutant *Salmonella* PhoP^c^, which has been used as a non-pathogenic *Salmonella* for intestinal inflammation studies. These observations raise concerns about using mutated *Salmonella* as a vector in cancer therapy. Further studies are needed to confirm the safety of these approaches for tumor therapy.

One limitation of this study is that PhoP^c^ is not wild-type pathogenic *Salmonella*. We had infected C57BL/6 mice with pathogenic *salmonella* SL1344 or SL14028. However, both strains caused death in over 90% of mice within 2 weeks of infection. Recent studies demonstrate that an early and rapid inflammatory response results in protection against the pathological effects of *S.* Typhimurium infection in Nramp1^+/+^ mice [35]. However, the C57BL/6 mice used in the current study were Nramp1^−/−^. Nramp1^+/+^ mice should be used to further understand the inflammatory response during pathogenic *Salmonella*-induced colitis in chronic inflammation.

In this study, we uncovered several novel aspects of *Salmonella* and effector AvrA: (1) we established a chronic *Salmonella* infection model to study inflammation, infection, and cancer; (2) we found that the AvrA level in *Salmonella* may alter its capacity of invasion and translocation; (3) we showed the biological effects of AvrA in inhibiting intestinal inflammation *in vivo*; and (4) we found that “non-pathogenic” PhoP^c^ induced less intestinal pathology but more liver abscess and gallbladder translocation. Infection, inflammation, and certain cancers are intimately linked [36], [37], [38]. In addition to providing insights into salmonellosis, an important and timely public health problem, studies using chronically *Salmonella*-infected mice could improve our understanding of the roles of bacteria in infectious diseases, inflammatory bowel diseases, and colitis-associated cancer.

## Materials and Methods

### Bacterial strains and growth condition

Bacterial strains used in this study included *Salmonella typhimurium* wild-type strain ATCC14028 (WT-SL) and *Salmonella* mutant strains PhoP^c^ [39], PhoP^c^AvrA^−^, and PhoP^c^AvrA^−^/AvrA^+^ (Table 3). Non-agitated microaerophilic bacterial cultures were prepared by inoculating 10 ml of Luria-Bertani broth with 0.01 ml of a stationary-phase culture followed by overnight incubation (∼18 h) at 37°C, as previously described [21].

**Table 3:** *Salmonella* strains used in this study.

Name	Description	References or source
PhoP^c^	Non-pathogenic complex regulator mutant derived from wild-type SL14028	Miller et al., 1990
PhoP^c^ AvrA−	AvrA^−^ mutation derived from PhoP^c^	Collier-Hyams *et al*., 2002
PhoP^c^AvrA−/AvrA+	PhoP^c^AvrA^−^ with complemented plasmid encoding AvrA	Collier-Hyams *et al*., 2002
SL1344	Wild-type *Salmonella* 1344 strain	Hardt et al., 1997 Provided by Dr. Galan
SB1117	SL 1344 with AvrA deletion	Hardt et al., 1997 Provided by Dr. Galan

### Streptomycin pre-treated mouse model

Animal experiments were performed by using specific pathogen–free female C57BL/6 mice (Taconic) that were 6–7 weeks old, as previously described [25]. Water and food were withdrawn 4 h before oral gavage with 7.5 mg/mouse of streptomycin (100 µl of sterile solution). Afterwards, animals were supplied with water and food *ad libitum*. Twenty hours after streptomycin treatment, water and food were withdrawn again for four hours before the mice were infected with 1×10^6^ CFU of *S. typhimurium* (100-µl suspension in HBSS) or treated with sterile HBSS (control) by oral gavage, as previously described [40]. At 1, 3, 6, 10, and 27 weeks after *Salmonella* infection, tissue samples were collected. If a mouse showed indications of aspirated fluid or significant body weight loss (10% or more), and did not die immediately, the mouse was humanely euthanized. All animals were handled in strict accordance with good animal practice as defined by the relevant national and/or local animal welfare bodies, and all animal work was approved by the University of Rochester University Committee on Animal Resources (UCAR) committee (UCAR 2007-065).

### Detection of *Salmonella* in intestine

Mouse feces (about 100 mg) were collected, vortexed in phosphate-buffered saline (PBS), and centrifuged for 10 min at 800 rpm. The sample was transferred to a clean microfuge tube and centrifuged again at 6000 rpm for 5 min. The supernatant was discarded, 200 µL PBS was added to the pellet, and it was vortexed. Intestinal *Salmonella* was detected by culturing fecal content at 37°C overnight on a BBL CHROMagar plate (BD Biosciences, San Jose, CA, USA). *Salmonella* species appeared mauve (rose to purple).

### Microbial analysis

After euthanasia, 1-ml samples from the cecal contents of colonized mice were taken and serially diluted in PBS. Total bacterial concentrations were determined by using counting chambers. *Salmonella* distribution was also determined by immunostaining.

### *Salmonella* burden in intestine, gallbladder, spleen, and liver

Intestine, gallbladder, spleen, and liver were dissected from each mouse, put into 14-ml tubes with 5 ml sterile PBS, cut into pieces with scissors, then homogenized adequately using a Polytron PT2100 (Kinematica, Switzerland). Each homogenate was diluted 1000× to 10,000× with LB, plated (100 µl) on MacConkey agar plates, and incubated at 37°C overnight. Colony-forming units were quantified.

### Histological testing

Tissues were fixed in 10% neutral buffered formaldehyde for 4 to 12 h, transferred into 70% ethanol, and processed by standard techniques. Sections (5 µm) were stained with hematoxylin and eosin (H & E). For immunostaining, antigens were retrieved by 10-min boiling in 10 mM citrate (pH 6.0). Blinded histologic inflammatory scores were performed by a validated scoring system [41]. *Salmonella* distribution in intestine was determined by immunostaining using anti–*Salmonella* lipopolysaccharide antibody (Santa Cruz Biotechnology, Santa Cruz, CA, USA).

### *Salmonella*-induced mouse cytokine secretion

Mouse blood samples were collected by cardiac puncture and placed in tubes containing EDTA (10 mg/ml) [20], [22]. Mouse cytokines were measured using a mouse cytokine 10-Plex Panel kit (Invitrogen, Carlsbad, CA, USA) according to the manufacturer's instructions. Briefly, beads of defined spectral properties were conjugated to protein-specific capture antibodies and added along with samples (including standards of known protein concentration, control samples, and test samples) into the wells of a filter-bottom microplate, where proteins bound to the capture antibodies over the course of a 2-hour incubation. After washing the beads, protein-specific biotinylated detector antibodies were added and incubated with the beads for 1 hour. After removal of excess biotinylated detector antibodies, the streptavidin-conjugated fluorescent protein R-phycoerythrin (streptavidin-RPE) was added and allowed to incubate for 30 minutes. After washing to remove unbound streptavidin-RPE, the beads were analyzed with the Luminex detection system (PerkinElmer CS1000 Autoplex Analyzer).

### Statistical analysis

Data are expressed as mean ± SD. All statistical tests were 2-sided. P values of less than .05 were considered to be statistically significant. For cecum data, four areas of mouse body were checked to obtain the average infection score. Then, multiple comparisons of mean infection score were performed using ANOVA. Following analysis of overall group differences on the mean scores at p≤.05 alpha levels were adjusted using the Tukey correction for multiple comparisons. As the study involved a longitudinal design, the generalized estimating equations (GEE) was utilized to provide statistical inference. The GEE approach is widely used due to its less stringent distributional assumptions and robustness properties, yielding valid inference regardless of the data distribution [42]. Thus, GEE provides more robust inference than the other popular generalized linear mixed-effects model, as the latter imposes analytic distributions that are often at odd with the distributions of data. Since, the pick-up mice for each experiment is random, we assume the loss of mice is completely random, the missing data issue does not consider. Therefore, the GEE procedure was used to assess treatment effects for each of the mouse weight outcomes. The analysis was conducted using the Genmod procedure in SAS. The overall treatment effects were modeled using the time by treatment interaction term through intake (week 0) to week 27. Statistical analyses were performed using SAS version 9.2 (SAS Institute, Inc., Cary, NC).
